# COVID-19: A Single-Center ICU Experience of the First Wave in the Philippines

**DOI:** 10.1155/2021/7510306

**Published:** 2021-01-30

**Authors:** Onion Gerald V. Ubaldo, Jose Emmanuel M. Palo, Jude Erric L. Cinco

**Affiliations:** Acute and Critical Care Institute, The Medical City–Ortigas, Pasig City 1605, Philippines

## Abstract

On January 30, 2020, the WHO declared the novel coronavirus of 2019 a pandemic, causing millions of cases and thousands of deaths worldwide, exposing the vulnerabilities of healthcare systems around the world with each country having its own experience. These ranged from patient clinical profiles to management recommendations and to government interventions. There is a paucity of published data regarding Philippine experience. This study is a retrospective, descriptive study of ninety-one COVID-19 probable patients admitted in the COVID ICU of The Medical City from March 16 to May 7, 2020. We described clinical and demographic characteristics amongst COVID-19-confirmed and -negative patients. Therapeutic interventions including COVID-19 investigational drug use and other organ failure strategies were noted and tested for association with ICU survivors and nonsurvivors. We observed that there was no therapeutic intervention that was associated with improved outcomes, with some interventions showing trends favoring the ICU nonsurvivor group. These interventions include, but are not limited to, the use of hydroxychloroquine and tocilizumab, and prone positioning. We also observed that a higher SAPS-3 score was associated with the COVID-19 positive group and the ICU nonsurvivor group. On PubMed search, there seems to be no Philippine-specific literature regarding COVID-19 ICU experience. Further investigations to include more variables are recommended.

## 1. Introduction

Clustering of cases presenting as pneumonia was reported in Wuhan Province, China, as early as November 2019 [1]. The cause for this was later identified as the new strain of coronavirus called the severe acute respiratory syndrome coronavirus 2 (SARS-CoV-2). These patients, all linked to Wuhan's Huanan Seafood Market, presented mostly with dry cough and fever. However, some patients required intensive care support for full-blown respiratory failure, leading to multiorgan failure and death. Since its discovery in China, SARS-CoV-2 has rapidly spread across the globe affecting millions. On January 30, 2020, the outbreak was declared a public health emergency of international concern by the World Health Organization (WHO) and a pandemic by March 11, 2020 [2].

On February 28, 83,562 confirmed coronavirus disease of 2019 (COVID-19) cases were reported, with only 4.5% being accounted for by countries outside of China [3, 4]. During the tail-end of February, incidence in China began to decrease, with Europe becoming the new epicenter of the virus. By March 10, 2020, Italy tallied more than 10,000 confirmed COVID-19 cases and by March 19, the country registered higher mortalities as compared to China [5]. The United States of America (USA) quickly followed suit tallying 124,655 new cases and 2,191 mortalities by March 29. The United Kingdom (UK) was not spared as they registered 17,089 confirmed COVID-19 cases and 1,019 fatalities by March 29 as well [5].

In the Philippines, the Department of Health (DOH) confirmed the first case of COVID-19 on January 30, 2020, and the first COVID-19-related mortality by February 2, 2020. The first locally transmitted case of COVID-19 was confirmed on March 7, 2020. As of May 25, 2020, the Philippines has a total of 14,319 confirmed cases, 873 fatalities, and 3,323 recoveries despite having community quarantine measures being instituted on March 15, 2020. This pandemic overwhelmed susceptible healthcare systems with the Philippines, considered as a developing country, being particularly vulnerable. International data regarding the COVID-19 experience have been ubiquitous while local Philippine data have been sparse. The Medical City (TMC), a tertiary, teaching, university-affiliated, hospital in the country, was at the forefront of the battle against COVID-19. We designed this study to describe patient characteristics, pre-admission laboratories, treatment strategies, and outcomes in terms of ICU survival or nonsurvival, as well as our experience in the frontlines, during the first influx of COVID-19 patients in the institution.

## 2. Materials and Methods

### 2.1. Population and Sample

We conducted a retrospective, descriptive study including all COVID-19 probable patients admitted in the TMC COVID Intensive Care Unit (ICU) from March 16 to May 7, 2020. A total of ninety-one patients were enrolled in this study which was approved by the Institutional Review Board (IRB) of TMC.

## 3. Methods

Ninety-one patients were admitted in the TMC COVID ICU from March 16 to May 7, 2020. These patients were enrolled in the study and their charts were reviewed.

Demographic data such as (1) age, (2) gender, and (3) SARS-CoV-2 status (either positive or negative) were collected. Severity of illness (SOI) scores were calculated using the Severe Acute Physiology Scoring 3 (SAPS-3). Laboratories on admission such as (1) hemoglobin (Hgb), (2) white blood cell (WBC) count, (3) platelet count, (4) lactate, and (5) PaO_2_/FiO_2_ ratios were recorded. Pre-admission comorbidities were grouped into (1) neurologic, (2) cardiac, (3) pulmonary, (4) renal, (5) endocrine, (6) autoimmune, and (7) malignancy, likewise noted. Therapeutic interventions used for the treatment of COVID-19 were also taken such as (1) use of hydroxychloroquine (HCQ), (2) use of tocilizumab (TCZ), (3) use of lopinavir/ritonavir, (4) use of intravenous immunoglobulin, (5) use of renal replacement therapy (RRT), (5) use of hemoperfusion (HA-330), (6) use of neuromuscular blocking agents (NMBA), (7) use of prone positioning, and (8) use of endotracheal intubation. Lastly, outcomes were recorded as (1) number of days intubated, (2) ICU survival, and (3) ICU nonsurvivors.

These data were recorded in the tabular form using a Microsoft© Excel file. All participants were de-identified and assigned control numbers for anonymity. The file was password-encrypted with access limited only to the authors. Data from the Excel file was then transferred to SPSS for further statistical analysis.

### 3.1. Analysis

Data collection and statistical analysis were done by the authors. General ICU statistical data are presented as total numbers and percentages when applicable. Demographics are presented in terms of means and standard deviations at a confidence interval of 95%. Percentages were also included. Initially, the participants were divided into two groups: (1) SARS-CoV-2 positive and (2) SARS-CoV-2 negative, to determine the differences between the two populations. Later, participants were then divided into two clinically significant outcome groups: (1) ICU survivors and (2) ICU nonsurvivors. Analysis was made between these outcomes and SARS-CoV-2-positive and -negative patients. The chi-square test for independence and Fisher's exact test were used for categorical variables, and the independent samples *t*-test was used for continuous variables after a test for normality was performed. A standardized mortality ratio (SMR) was computed for the totality of the COVID ICU, for the COVID-19 confirmed patients, and the non-COVID-19 patients. A *p* value of <0.05 was used as a threshold for statistical significance. SPSS 21.0 (Copyright © SPSS Inc. 1989–2007) was used for all statistical analyses done.

## 4. Results and Discussion

### 4.1. Results

A total of 91 patients were admitted in the TMC COVID ICU and subsequently enrolled, of which 31 patients turned out to be SARS-CoV-2 positive (34%) while 60 patients were negative (66%). The average age of the enrolled participants was 65 years with a standard deviation of 14 years, with more than half being male (62.6%). Among all the enrolled participants, a total of 63 patients were intubated (69.2%), of which 17 patients (27%) were successfully extubated. Meanwhile, a total of 29 SARS-CoV-2-confirmed patients were intubated (93%) and only 6 among them were successfully extubated (21%). The cumulative mortality rate of all admissions was 40.7% (*n* = 37), while the COVID-19-specific mortality rate was 71% (*n* = 22) (Table 1).

Table 2 shows a comparison of variables between the SARS-CoV-2-positive and -negative groups. In terms of demographic variables, the SARS-CoV-2-positive group had a higher mean SOI score and had fewer patients of the male gender. There was no statistically detectable significant difference between the two groups. As for preexisting comorbidities prior to ICU admission, most SARS-CoV-2-positive patients had cardiac comorbidities. However, only renal comorbidity seems to exhibit a statistically detectable significance (*p* < 0.001) with an absolute percentage difference of 39.5% between the two groups, favoring the SARS-CoV-2-negative group. In terms of pre-admission laboratory parameters, higher hemoglobin levels (*p*=0.002), lower WBC counts (*p*=0.003), and lower PF ratios (*p*=0.002) were observed among the SARS-CoV-2-positive group. Our calculated total SMR was 1.65, with the usual institutional ICU SMR at 0.4. The SARS-CoV-2-confirmed and -negative SMR was 2.34 and 1.15, respectively.

A comparison between variables and the clinically significant outcome groups (ICU survival and nonsurvival) is presented in Table 3. It was observed that only the SOI and SARS-CoV-2 status exhibit statistically detectable difference (*p* < 0.001for both). ICU nonsurvivors were more likely to have higher SAPS-3 scores (mean of 60 with a standard deviation of 17), and they were more likely to have a positive SARS-CoV-2 result with an absolute percentage difference of 42.8%. Same with Table 2, most ICU nonsurvivors had cardiac comorbidities but no pre-admission comorbidity appears to be associated with the outcome measures. The nonsurvivor group was associated with lower PF ratios (mean 166, standard deviation 99) as compared to the survivor group (mean 258, standard deviation 145). Although hemoglobin levels and WBC count did not exhibit statistical significance, there was a trend towards favoring the ICU mortality group if hemoglobin levels were higher and WBC counts were lower. Seven out of the nine therapeutic strategy variables exhibited statistically detectable difference, namely, use of HCQ, use of TCZ, use of lopinavir/ritonavir, use of CRRT, use of NMBA, use of prone positioning, and use of endotracheal intubation. It was observed that use of these interventions favored being included in the ICU nonsurvivor group.

Table 4 shows a comparison between the ICU survivors and nonsurvivors amongst the confirmed COVID-19 patients only. It was observed that the nonsurvivor group had higher SAPS-3 scores as compared to the survivor group (absolute difference 17.6, *p*=0.008). Similar to the results seen in Table 3, there seems to be no pre-admission comorbidity associated with the outcome groups. Pre-admission lactate level exhibited statistically significant difference (1 mmol/L vs. 2.2 mmol/L for the survivor and nonsurvivor group, respectively,*p*=0.021), showing that a higher lactate is associated with ICU nonsurvivors. The statistical significance of PF ratio, which was previously observed in Table 3, is not seen. Most of the treatment strategies used among the COVID-19-confirmed participants were not associated with any outcome group aside from the use of CRRT (*p* < 0.001) and endotracheal intubation (*p* < 0.001), with their use being associated with those belonging to the ICU nonsurvivor group. Among those who were intubated, the ICU nonsurvivor group seems to have a shorter time of being on the mechanical ventilator probably because of a shorter time of being alive (13 days for the survivor group and 8 days for the nonsurvivor group, *p*=0.005).

Finally, comparison between ICU survivors and nonsurvivors amongst the COVID-19-negative patients is shown in Table 5. It was observed that the ICU nonsurvivor group was younger (mean 57 years old, standard deviation 14), had more patients of the male gender (80%), and had higher SAPS-3 scores (mean 58.7, standard deviation 20.1) as compared to the ICU survivor group. Only age exhibited statistically significant difference (*p*=0.015), but a higher SAPS-3 shows a trend favoring the ICU nonsurvivor group. Having any neurologic comorbidity was the only variable seen among pre-admission comorbidities that exhibited statistically detectable difference (*p*=0.026). Among COVID-19-negative patients, there seems to be no laboratory parameter that exhibited statistically detectable difference in contrast to what the previous tables showed. Furthermore, among COVID-19-negative patients, only the use of HCQ and use of endotracheal intubation showed statistically detectable difference (*p*=0.036 and *p*=0.001, respectively), with the absence of their use favoring the ICU survivor group.

### 4.2. Discussion

In this retrospective, observational, single-center experience in a tertiary hospital in the Philippines, we observed that there was no therapeutic strategy associated with ICU survival amongst patients admitted for probable COVID-19 in the COVID ICU. This observation was also seen despite having the participants classified into confirmed COVID-19 and negative for COVID-19 patients. It was also consistent that having a high pre-admission SAPS-3 score was associated with ICU nonsurvival.

These findings are parallel to the current literature and the worldwide milieu with COVID-19, where most countries are still actively searching for the specific drug or intervention, short of a vaccine, that may help reduce COVID-19-related mortality and improve clinically significant outcomes [4,6–9]. During this study, there were local recommendations favoring the use HCQ, TCZ, and lopinavir/ritonavir in the clinical management of COVID-19 patients based on case reports/series and small randomized clinical controlled trials. As of the writing of this manuscript, more robust literature was published proving otherwise [10]. These latter trials appear to be more congruent with our findings. In our single-center experience, we saw variation in how these investigational drugs were initiated, which could explain the lack of survival benefit seen in our study. These medications, TCZ in particular, should be used before the onset of the COVID-19-related cytokine storm in order to be effective [11,12].

Most of our COVID ICU admissions were due to respiratory failure requiring endotracheal intubation since our threshold to intubate was low because of the impending dread of aerosolizing the virus as our institution had limited negative isolation rooms [13]. This is seen with our total intubation rate of 69.2% and our SARS-CoV-2 intubation rate of 93%. Currently, COVID-19-related recommendations have been consistent on attempting to use noninvasive means (i.e., high flow nasal cannula) to oxygenate but not to otherwise delay endotracheal intubation once clinically indicated [3,8,14]. Most deterioration was after 48 hours due to either refractory hypoxemia, which may or may not be acute respiratory distress syndrome (ARDS) related, or refractory shock, both of which were most certainly related to the progressive and relentless nature of the disease. Our institution's standard of care is to use evidence-based ARDS management interventions such as prone positioning and the low tidal volume-high PEEP strategy. We extrapolated these interventions into caring for the mechanically ventilated COVID-19 patients as there was contradicting literature available at that time [15,16].

Despite these efforts, our COVID-19 extubation rate was only at 21%. The noneffectiveness of these interventions is reflected by the absence of a survival benefit for both in the COVID-19-confirmed and -negative subgroup. It was also observed that the number of days intubated was significantly lesser for the ICU nonsurvivor group than that for the nonsurvivor group for both COVID-19 and non-COVID patients. We postulate that this difference is because the surviving group was more days alive than the nonsurviving group.

Apart from mechanical ventilation practices, we also adjusted our sedation processes. Initially, we were highly dependent on propofol, fentanyl, and midazolam. For several days, we kept the patients deeply sedated with a Richmond-Agitation-Sedation-Score (RASS) of −4 to −5 to reduce the risk for self-induced lung injury (SILI) and negative pressure pulmonary edema [13,14]. We noticed that, after weeks of being mechanically ventilated, most patients failed spontaneous awakening trials (SAT) despite passing spontaneous breathing trials (SBT). This could explain why our patients were on the mechanical ventilator longer. Our consideration was that we used excessive amounts of benzodiazepines and opioids, especially that most of our patients had renal failure requiring RRT.

We then shifted to a nonopioid, nonbenzodiazepine strategy using propofol plus ketamine since the latter has immunomodulatory effects by decreasing IL-6 levels [17], akin to TCZ. To lessen the time on propofol and ketamine, we started to overlap with atypical anti-psychotics (i.e., quetiapine and risperidone) and dexmedetomidine. To reduce the cumulative dose of opioids used, we used pregabalin and gabapentin to augment our pain control measures. It was only then that we were able to extubate some of our patients.

Approximately 30.8% of our enrolled participants underwent CRRT and 8 patients used hemoperfusion, theoretically, to tide them over the COVID-19 cytokine storm [18]. As with the other interventions seen, it proved to be nonbeneficial to both confirmed COVID-19 and non-COVID-19 patients. Our institution's nephrology group drafted institution-specific guidelines on initiating RRT and hemoperfusion using the HA-330 disposable cartridge© (Jafron Biomedical Co, Ltd., Zhuhai City, China). However, hemoperfusion will not be initiated if stocks were not enough for three consecutive doses. Furthermore, CRRT machines were also of limited supply and RRT had to be carefully coordinated. The usual CRRT duration of 24–48 hours was decreased to 12 hours for consecutive days to accommodate as many patients needing the therapy as possible. We had to “ration” our supplies to be able to serve the greatest number of patients.

Our institution paralleled early experiences of the Chinese and the Italians during the influx of probable COVID patients in the ICU. At the start of the influx, we were overwhelmed by the volume of patients coming in. Most of our healthcare staff became symptomatic necessitating at least 14 days of quarantine and thinned out our workforce. Most of the critically ill patients had stayed longer in the emergency department (ED) while waiting for an available ICU bed. Inasmuch as ED personnel are competent, they were not trained to handle severely ill patients of that quantity; hence, interventions (i.e., prone positioning, paralysis, and RRT) were delayed and were only facilitated once the patients were sent up to the ICU, which could be as long as 72 hours from presentation. These could also explain why our mortality rate was as such.

As a response, we augmented our surge capacity by converting the nonnegative pressure ICU beds into negative pressure ICU beds bringing our COVID ICU bed capacity from 3 beds to 16 beds. An additional five beds in the COVID-19 dedicated regular nursing unit was converted to telemeter capable units also able to handle critically ill patients. The nursing staff were also amplified by re-assigning additional non-ICU staff to the ICU. These new recruits were trained and supervised by the senior ICU staff and the intensivists. Barriers were implemented to separate patients, and air purifiers with high efficiency particulate air (HEPA) filters were placed in the ED so that we could optimize the use of our high flow nasal cannulas.

Because of resource allocation, which includes healthcare staff, elective surgeries were suspended and restricted to emergency cases (i.e., large bowel perforations and obstructions, lower gastrointestinal bleeding, and complicated diverticulitis). The increase in COVID-19 cases locally led to an adjustment of previously used surgical techniques to better protect the surgical team and the next patient to use to theater from getting infected, especially during aerosol generating medical procedures. Open surgical techniques were preferred over minimally invasive procedures to reduce risk of aerosolization and decrease operative time [19]. If possible, nonemergent cases were given optimal medical management and closely followed up via phone and video calls [19,20]. Finally, ancillary radiologic procedures were also frequently used, as both a diagnostic and a therapeutic technique, to reduce amounts of surgeries [20].

Furthermore, in order to protect the surgical team, all patients for surgery (including elective tracheostomies for mechanical ventilator dependent patients unable to be weaned off) were tested for COVID-19, appropriate personal protective equipment (PPE) was used, and air cleaning for at least 30 minutes after the aerosol generating procedure was done prior to the next surgical case, in line with international precedent [21]. However, the increase in protection with the use of PPE was reported to have some adverse effects in both technical and nontechnical skills (i.e., communication) for the surgical team [21], the description of which is beyond the scope of this research.

**Figure 1:**
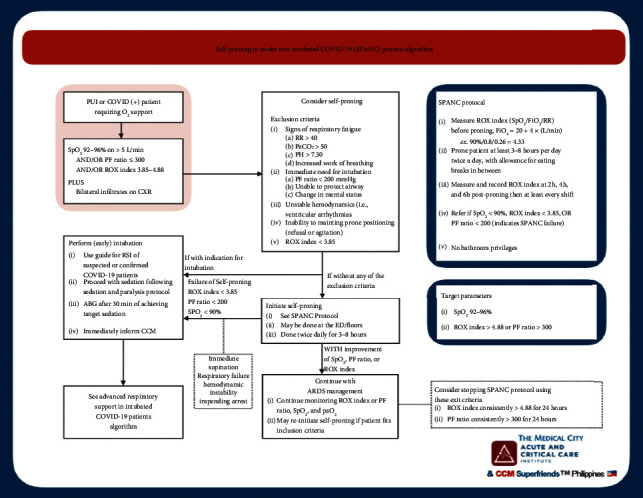
Self-Proning in Awake Nonintubated COVID-19 (SPANC) patients protocol.

**Figure 2:**
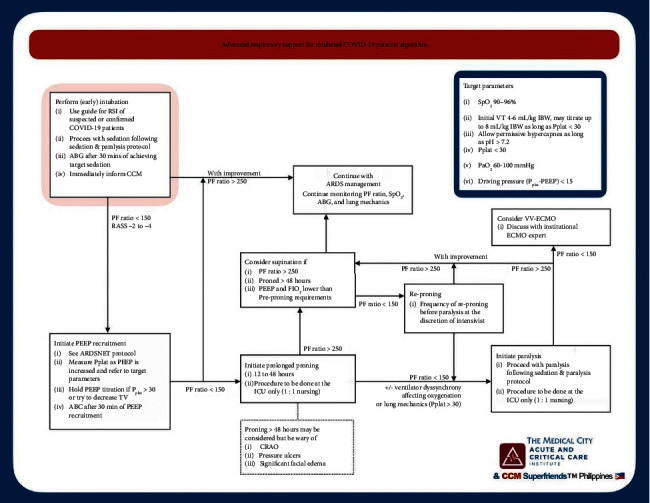
Advanced respiratory support for intubated COVID-19 patients algorithm.

A group of local critical care physicians developed protocols, based on best evidence and practice [22,23], in an attempt to curb the influx of patients being admitted in the ICU. The Self-Proning in Awake Nonintubated COVID-19 (SPANC) patients protocol was instituted to decrease the number of intubations that was threatening the supply of mechanical ventilators (see Figure 1). The group also created an advanced mechanical ventilation protocol for COVID-19 patients in order to streamline ventilation settings towards best practices as well (see Figure 2).

Even with our best efforts, our ICU showed an increase in our SMR from a previous average of 0.4 to 1.65. This increase could be attributed to the high mortality experienced in the confirmed COVID-19 group which pulled our total SMR upwards, with an SMR of 2.34 for the COVID-19 group compared to an SMR of 1.15 for the non-COVID group. We feel that the high SMR was a reflection of the “strain” the pandemic put on our healthcare system, that even though some individuals put in “heroic” efforts, these were not enough, and institutional changes were necessary to cope with the influx.

In this study, we did not include critically ill, COVID-19 probable patients that were not admitted in the COVID ICU. Some patients were admitted in the converted telemetry units in the COVID-19 dedicated regular nursing unit while some stayed in the emergency department. Doses of vasopressors and sedatives were not included, as well as secondary infections, analysis on the use of noninvasive ventilation, adverse events related to the therapeutic strategies, and cause of demise. For the purposes of this study, we decided to focus on the use of investigational medications/procedures for the treatment of COVID-19 and understand their associations with clinically significant outcomes. Perhaps, these are avenues for future research endeavors using the same population and design.

## 5. Conclusions

In conclusion, amongst critically ill COVID-19 probable patients admitted in the TMC COVID ICU from March 16 to May 7, 2020, there appears to be no survival benefit among the therapeutic interventions used, with some interventions having a trend towards inclusion in the nonsurvivor group. To date, COVID-19 Philippine literature is limited to case reports/series, mathematical projections, and mental health [24]. There is a lack of data regarding experiences, both clinical management and administrative, in dealing with this pandemic. This study contributes significant COVID-19-related literature and may help to deal with an influx of cases in the future. As further literature becomes available, our current practices will continue to be in flux and evolve. A further study to include additional variables is recommended.

## Figures and Tables

**Table 1:** Descriptive statistics.

Total number of patients admitted	91
Total number of SARS-CoV-2-confirmed patients	31
Case positive rate	34%
Total number of SARS-CoV-2-negative patients	60
Case negative rate	66%
Total number of patients who were intubated	63
Intubation rate	69.2%
Total number of patients who were successfully extubated	17
Successful extubation rate	27%
Total number of SARS-CoV-2-confirmed patients who were intubated	29
SARS-CoV-2 intubation rate	93%
Total number of SARS-CoV-2 patients who were successfully extubated	6
SARS-CoV-2 extubation rate	21%
Total number of SARS-CoV-2-negative patients who were intubated	26
SARS-CoV-2-negative intubation rate	57%
Total number of SARS-CoV-2-negative patients who were successfully extubated	11
SARS-CoV-2-negative extubation rate	42%
Total number of mortalities	37
Cumulative mortality rate	40.7%
Total number of SARS-CoV-2-confirmed patients who died	22
Case positive mortality rate	71%
Total number of SARS-CoV-2-negative patients who died	15
Case negative mortality rate	25%

**Table 2:** Variables and SARS-CoV-2 status.

	SARS-CoV-2 positive (*n* = 31)	SARS-CoV-2 negative (*n* = 60)	Significance (two-tailed)
Demographics			
Age (years)	65 (±12)	65 (±15)	0.918
Gender (% of males)	58.1%	65%	0.648
SAPS-3	56.5 (±16.3)	50.6 (±15.5)	0.102
Comorbidities			
Neurologic			0.078
Yes	2 (6.5%)	13 (21.7%)	
No	29 (93.5%)	47 (78.3%)	
Cardiovascular			0.112
Yes	21 (67.7%)	50 (83.3%)	
No	10 (32.3%)	10 (16.7%)	
Pulmonary			0.061
Yes	6 (19.4%)	24 (40%)	
No	25 (80.6%)	36 (60%)	
Renal			<0.001
Yes	2 (6.5%)	27 (45%)	
No	29 (93.5%)	33 (55%)	
Endocrine			0.657
Yes	15 (48.4%)	25 (41.7%)	
No	16 (51.6%)	35 (58.3%)	
Autoimmune			0.713
Yes	2 (6.5%)	7 (11.7%)	
No	29 (93.5%)	53 (88.3%)	
Malignancy			0.320
Yes	2 (6.5%)	9 (15%)	
No	29 (93.5%)	51 (85%)	
Laboratories on ICU admission			
Hemoglobin (g/L)	135 (±17)	120 (±31)	0.002
WBC count (×10^3^/*µ*L)	8.5 (±4.6)	13 (±9)	0.003
Platelets (×10^9^/L)	220 (±75)	238 (±109)	0.382
PF ratio	163 (±103)	255 (±141)	0.002
Lactate (mmoL/L)	2 (±2)	2.9 (±2)	0.113
Treatment strategy			
Hydroxychloroquine			<0.001
Yes	25 (80.6%)	9 (15%)	
No	6 (19.4%)	51 (85%)	
Tocilizumab			<0.001
Yes	17 (54.8%)	2 (3.3%)	
No	14 (45.2%)	68 (96.7%)	
Lopinavir/ritonavir			0.006
Yes	6 (19.4%)	1 (1.7%)	
No	25 (80.6%)	59 (98.3%)	
CRRT			<0.001
Yes	18 (58.1%)	10 (16.7%)	
No	13 (41.9%)	50 (83.3%)	
NMBA			0.017
Yes	6 (19.4%)	2 (3.3%)	
No	25 (80.6%)	58 (96.7%)	
Prone positioning			<0.001
Yes	13 (41.9%)	3 (5%)	
No	18 (58.1%)	57 (95%)	
IVIG			0.037
Yes	3 (9.7%)	0	
No	28 (90.3%)	60 (100%)	
HA-330			<0.001
Yes	8 (25.8%)	0	
No	23 (74.2%)	60 (100%)	
Intubation			<0.001
Yes	29 (93.5%)	34 (56.7%)	
No	2 (6.5%)	26 (43.3%)	
Outcomes			<0.001
ICU survivor	9 (29%)	45 (75%)	
ICU mortality	22 (71%)	15 (25%)	
Number of days intubated	13 (±7)	8 (±7)	0.005
Standardized mortality ratio	2.34	1.15	

**Table 3:** Variables and outcomes.

	ICU survivors (*n* = 54)	ICU nonsurvivors (*n* = 37)	Significance
Demographics			
Age (years)	66 (±15)	63 (±13)	0.253
Gender (% of males)	61.1%	64.9%	0.826
SAPS-3	47 (±13)	60 (±17)	<0.001
SARS-CoV-2 status			<0.001
Positive	9 (16.7%)	22 (59.5%)	
Negative	45 (83.3%)	15 (40.5%)	
Comorbidities			
Neurologic			0.003
Yes	14 (25.9%)	1 (2.7%)	
No	40 (74.1%)	36 (97.3%)	
Cardiovascular			0.440
Yes	44 (81.5%)	27 (73%)	
No	10 (18.5%)	10 (27%)	
Pulmonary			0.821
Yes	17 (31.5%)	13 (35.1%)	
No	37 (68.5%)	24 (64.9%)	
Renal			0.039
Yes	22 (40.7%)	7 (18.9%)	
No	32 (59.3%)	30 (81.1%)	
Endocrine			0.831
Yes	23 (42.6%)	17 (45.9%)	
No	31 (57.4%)	20 (54.1%)	
Autoimmune			0.302
Yes	7 (13%)	2 (5.4%)	
No	47 (87%)	35 (94.6%)	
Malignancy			0.344
Yes	5 (9.3%)	6 (16.2%)	
No	49 (90.7%)	31 (83.8%)	
Laboratories on ICU admission			
Hemoglobin (g/L)	120 (±30)	131 (±23)	0.050
WBC count (×10^3^/*µ*L)	12.7 (±10)	9.5 (±5)	0.081
Platelets (×10^9^/L)	231 (±91)	232 (±110)	0.952
PF ratio	258 (±145)	166 (±99)	0.003
Lactate (mmol/L)	2.7 (±2.2)	2.4 (±1.8)	0.537
Treatment strategy			
Hydroxychloroquine			<0.001
Yes	12 (22.2%)	22 (59.5%)	
No	42 (77.8%)	15 (40.5%)	
Tocilizumab			0.008
Yes	6 (11.1%)	13 (35.1%)	
No	48 (88.9%)	24 (64.9%)	
Lopinavir/ritonavir			0.017
Yes	1 (1.9%)	6 (16.2%)	
No	53 (98.1%)	31 (83.8%)	
CRRT			<0.001
Yes	8 (14.8%)	20 (54.1%)	
No	46 (85.2%)	17 (45.9%)	
NMBA			0.007
Yes	1 (1.9%)	7 (18.9%)	
No	53 (98.1%)	30 (81.1%)	
Prone positioning			0.023
Yes	4 (7.4%)	12 (32.4%)	
No	50 (92.6%)	25 (67.6%)	
IVIG			0.564
Yes	1 (1.9%)	2 (5.4%)	
No	53 (98.1%)	35 (94.6%)	
HA-330			0.058
Yes	2 (3.7%)	6 (16.2%)	
No	52 (96.3%)	31 (83.6%)	
Intubation			<0.001
Yes	27 (50%)	36 (97.3%)	
No	27 (50%)	1 (2.7%)	
Others			
Number of days intubated	12 (±8)	9 (±7)	0.111

**Table 4:** Variables and outcomes among COVID-19-confirmed patients.

	ICU survivors (*n* = 9)	ICU nonsurvivors (*n* = 22)	*p* value (two-tailed)
Demographics			
Age (years)	59 (±12)	67 (±12)	0.151
Gender (% of males)	66.7%	54.5%	0.696
SAPS-3	44 (±14.7)	61.6 (±14.3)	0.008
Comorbidities			
Neurologic			0.503
Yes	1 (11.1%)	1 (4.5%)	
No	8 (88.9%)	21 (95.5%)	
Cardiovascular			0.417
Yes	5 (55.6%)	16 (72.7%)	
No	4 (44.4%)	6 (27.3%)	
Pulmonary			0.642
Yes	1 (11.1%)	5 (22.7%)	
No	8 (88.9%)	17 (80.6%)	
Renal			1.000
Yes	0	2 (9.1%)	
No	9 (100%)	20 (90.9%)	
Endocrine			0.433
Yes	3 (33.3%)	12 (54.5%)	
No	6 (66.7%)	10 (45.5%)	
Autoimmune			0.077
Yes	2 (22.2%)	0	
No	7 (77.8%)	22 (100%)	
Malignancy			1.000
Yes	0	2 (9.1%)	
No	9 (100%)	20 (90.9%)	
Laboratories on ICU admission			
Hemoglobin (g/L)	134 (±21)	135 (±15)	0.806
WBC count (×10^3^/*µ*L)	8.5 (±6.2)	8.4 (±4)	0.997
Platelets (×10^9^/L)	259 (±109)	204 (±50)	0.179
PF ratio	201 (±152)	148 (±73)	0.344
Lactate (mmol/L)	1 (±2.5)	2.2 (±2)	0.021
Treatment strategy			
Hydroxychloroquine			0.642
Yes	8 (88.9%)	17 (77.3%)	
No	1 (11.1%)	5 (22.7%)	
Tocilizumab			0.456
Yes	6 (66.7%)	11 (50%)	
No	3 (33.3%)	11 (50%)	
Lopinavir/ritonavir			0.642
Yes	1 (11.1%)	5 (22.7%)	1
No	8 (88.9%)	17 (77.3%)	
CRRT			0.017
Yes	2 (22.2%)	16 (72.7%)	
No	7 (77.8%)	6 (27.3%)	
NMBA			0.642
Yes	1 (11.1%)	5 (22.7%)	
No	8 (88.9%)	17 (77.3%)	
Prone positioning			0.696
Yes	3 (33.3%)	10 (45.5%)	
No	6 (66.7%)	12 (54.5%)	
IVIG			1.000
Yes	1 (11.1%)	2 (9.1%)	
No	8 (88.9%)	20 (90.9%)	
HA-330			1.000
Yes	2 (22.2%)	6 (27.3%)	
No	7 (77.8%)	16 (72.7%)	
Intubation			0.077
Yes	7 (77.8%)	22 (100%)	
No	2 (22.2%)	0	
Others			
Number of days intubated	19 (±7)	12 (±6)	0.048

**Table 5:** Variables and outcomes among non-COVID-19 patients.

	ICU survivors (*n* = 45)	ICU nonsurvivors (*n* = 15)	*p* value (two-tailed)
Demographics			
Age (years)	68 (±15)	57 (±14)	0.015
Gender (% of males)	60%	80%	0.218
SAPS-3	47.8 (±12.8)	58.7 (±20.1)	0.066
Comorbidities			
Neurologic			0.026
Yes	13 (28.9%)	0	
No	32 (71.1%)	15 (100%)	
Cardiovascular			0.250
Yes	39 (86.7%)	11 (73.3%)	
No	6 (13.3%)	4 (26.7%)	
Pulmonary			0.242
Yes	16 (35.6%)	8 (53.3%)	
No	29 (64.4%)	7 (46.7%)	
Renal			0.375
Yes	22 (48.9%)	5 (33.3%)	
No	23 (51.1%)	10 (66.7%)	
Endocrine			0.552
Yes	20 (44.4%)	5 (33.3%)	
No	25 (55.6%)	10 (66.7%)	
Autoimmune			1.000
Yes	5 (11.1%)	2 (13.3%)	
No	40 (88.9%)	13 (86.7%)	
Malignancy			0.208
Yes	5 (11.1%)	4 (26.7%)	
No	40 (88.9%)	11 (73.3%)	
Laboratories on ICU admission			
Hemoglobin (g/L)	118 (±31)	126 (±30)	0.358
WBC count (×10^3^/*µ*L)	13.6 (±10)	11.3 (±7)	0.328
Platelets (×10^9^/L)	226 (±87)	274 (±157)	0.275
PF ratio	273 (±142)	203 (±133)	0.154
Lactate (mmoL/L)	2.9 (±2.2)	2.8 (±1.6)	0.948
Treatment strategy			
Hydroxychloroquine			0.036
Yes	4 (8.9%)	5 (33.3%)	
No	41 (91.1%)	10 (66.7%)	
Tocilizumab			0.059
Yes	0	2 (13.3%)	
No	45 (100%)	13 (86.7%)	
Lopinavir/ritonavir			0.250
Yes	0	1 (6.7%)	
No	45 (100%)	14 (93.3%)	
CRRT			0.250
Yes	6 (13.3%)	4 (26.7%)	
No	39 (86.7%)	11 (73.3%)	
NMBA			0.059
Yes	0	2 (13.3%)	
No	45 (100%)	13 (86.7%)	
Prone positioning			0.151
Yes	1 (2.2%)	2 (13.3%)	
No	44 (97.8%)	13 (86.7%)	
IVIG			—
Yes	0	0	
No	45 (100%)	15 (100%)	
HA-330			—
Yes	0	0	
No	45 (100%)	15 (100%)	
Intubation			0.001
Yes	20 (44.4%)	14 (93.3%)	
No	25 (55.6%)	1 (6.7%)	
Others			
Number of days intubated	10 (±7)	5 (±5)	0.036

## Data Availability

Data and datasets that were used in this study are available upon request to Dr. Onion Gerald V. Ubaldo at onion.ubaldo@gmail.com.
